# HMG CoA reductase inhibitors (statins) for people with chronic kidney disease not requiring dialysis

**DOI:** 10.1590/1516-3180.20141325T1

**Published:** 2014-07-22

**Authors:** Suetonia C. Palmer, Sankar D. Navaneethan, Jonathan C. Craig, David D. Johnson, Vlado Perkovic, Jorgen Hegbrant, Giovanni F. M. Strippoli

## Abstract

**BACKGROUND:**

Cardiovascular disease (CVD) is the most frequent cause of death in people with early stages of chronic kidney disease (CKD), for whom the absolute risk of cardiovascular events is similar to people who have existing coronary artery disease. This is an update of a review published in 2009, and includes evidence from 27 new studies (25,068 participants) in addition to the 26 studies (20,324 participants) assessed previously; and excludes three previously included studies (107 participants). This updated review includes 50 studies (45,285 participants); of these 38 (37,274 participants) were meta-analysed.

**OBJECTIVE:**

To evaluate the benefits (such as reductions in all-cause and cardiovascular mortality, major cardiovascular events, MI and stroke; and slow progression of CKD to end-stage kidney disease (ESKD)) and harms (muscle and liver dysfunction, withdrawal, and cancer) of statins compared with placebo, no treatment, standard care or another statin in adults with CKD **who were not on dialysis.**

**METHODS:**

*Search methods:* We searched the Cochrane Renal Group's Specialised Register to 5 June 2012 through contact with the Trials' Search Co-ordinator using search terms relevant to this review. *Selection criteria:* Randomised controlled trials (RCTs) and quasi-RCTs that compared the effects of statins with placebo, no treatment, standard care, or other statins, on mortality, cardiovascular events, kidney function, toxicity, and lipid levels in adults with CKD not on dialysis were the focus of our literature searches. *Data collection and analysis:* Two or more authors independently extracted data and assessed study risk of bias. Treatment effects were expressed as mean difference (MD) for continuous outcomes (lipids, creatinine clearance and proteinuria) and risk ratio (RR) for dichotomous outcomes (major cardiovascular events, all-cause mortality, cardiovascular mortality, fatal or non-fatal myocardial infarction (MI), fatal or non-fatal stroke, ESKD, elevated liver enzymes, rhabdomyolysis, cancer and withdrawal rates) with 95% confidence intervals (CI).

**MAIN RESULTS:**

We included 50 studies (45,285 participants): 47 studies (39,820 participants) compared statins with placebo or no treatment and three studies (5547 participants) compared two different statin regimens in adults with CKD who were not yet on dialysis. We were able to meta-analyse 38 studies (37,274 participants).

The risk of bias in the included studies was high. Seven studies comparing statins with placebo or no treatment had lower risk of bias overall; and were conducted according to published protocols, outcomes were adjudicated by a committee, specified outcomes were reported, and analyses were conducted using intention-to-treat methods. In placebo or no treatment controlled studies, adverse events were reported in 32 studies (68%) and systematically evaluated in 16 studies (34%).

Compared with placebo, statin therapy consistently prevented major cardiovascular events (13 studies, 36,033 participants; RR 0.72, 95% CI 0.66 to 0.79), all-cause mortality (10 studies, 28,276 participants; RR 0.79, 95% CI 0.69 to 0.91), cardiovascular death (7 studies, 19,059 participants; RR 0.77, 95% CI 0.69 to 0.87) and MI (8 studies, 9018 participants; RR 0.55, 95% CI 0.42 to 0.72). Statins had uncertain effects on stroke (5 studies, 8658 participants; RR 0.62, 95% CI 0.35 to 1.12).

Potential harms from statin therapy were limited by lack of systematic reporting and were uncertain in analyses that had few events: elevated creatine kinase (7 studies, 4514 participants; RR 0.84, 95% CI 0.20 to 3.48), liver function abnormalities (7 studies, RR 0.76, 95% CI 0.39 to 1.50), withdrawal due to adverse events (13 studies, 4219 participants; RR 1.16, 95% CI 0.84 to 1.60), and cancer (2 studies, 5581 participants; RR 1.03, 95% CI 0.82 to 130).

Statins had uncertain effects on progression of CKD. Data for relative effects of intensive cholesterol lowering in people with early stages of kidney disease were sparse. Statins clearly reduced risks of death, major cardiovascular events, and MI in people with CKD who did not have CVD at baseline (primary prevention).

**AUTHORS' CONCLUSIONS:**

Statins consistently lower death and major cardiovascular events by 20% in people with CKD not requiring dialysis. Statin-related effects on stroke and kidney function were found to be uncertain and adverse effects of treatment are incompletely understood. Statins have an important role in primary prevention of cardiovascular events and mortality in people who have CKD.

**This is the abstract of a Cochrane Review published in the Cochrane Database of Systematic Reviews 2014, issue 5, Art. No.: CD007784. DOI:** 10.1002/14651858.CD007784.pub2 (http://onlinelibrary.wiley.com/doi/10.1002/14651858.CD007784.pub2/abstract). For full citation and authors' details, see reference 1.
